# Molecular characterization of *Cryptosporidium* and *Enterocytozoon bieneusi* in Père David's deer (*Elaphurus davidianus*) from Shishou, China

**DOI:** 10.1016/j.ijppaw.2019.09.001

**Published:** 2019-09-05

**Authors:** Fujie Xie, Zhenjie Zhang, Aiyun Zhao, Bo Jing, Meng Qi, Rongjun Wang

**Affiliations:** a College of Animal Science, Tarim University, Alar, Xinjiang, 843300, China; b College of Animal Science and Veterinary Medicine, Henan Agricultural University, Zhengzhou, Henan, 450002, China

**Keywords:** *Cryptosporidium*, *Enterocytozoon bieneusi*, Genotype, SSU rRNA, Père David's deer

## Abstract

*Cryptosporidium* and *Enterocytozoon bieneusi* are important intestinal pathogens that infect humans and various animals. Few reports are available regarding the infections of the two pathogens in Père David's deer. In this study, polymerase chain reaction (PCR) confirmed *Cryptosporidium* infection in two (1.6%) and *E. bieneusi* in 45 (35.2%) of 128 fecal samples collected from Père David's deer in the National Nature Reserve of Shishou, Hubei Province, China. *C. parvum* (n = 1) and *Cryptosporidium* deer genotype (n = 1) were identified using the small subunit rRNA (SSU rRNA) gene. The *C. parvum* was further subtyped as IIdA20G1 by sequencing analysis of the 60-kDa glycoprotein (gp60) gene. The identity of *E. bieneusi* was confirmed by an internal transcribed spacer (ITS) gene; the HLJD-V (n = 42) and MWC_d1 (n = 3) genotypes were identified, with the former clustering in group 2 and the latter in group 1. These data suggest that the Père David's deer were infected with host-specific and/or zoonotic genotypes of these pathogens, implicating Père David's deer could be a potential source of human *Cryptosporidium* infection.

## Highlights

• *C. parvum* subtype IIdA20G1 was firstly identified in Pere David's deer.
• Genotypes HLJD-V and MWC_d1 were detected from 45 *E. bieneusi*-positive samples.
• The present study implicated Père David's deer could be a potential source of human infection.

## Graphical abstract

**Figure:**
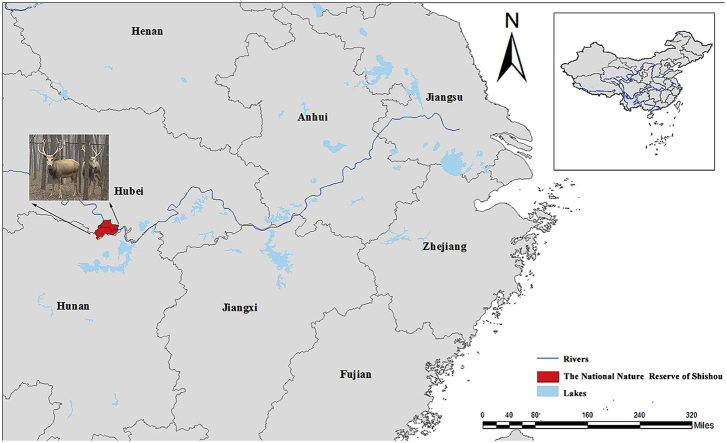

## 1 Introduction

*Cryptosporidium* spp. and *Enterocytozoon bieneusi* infections can cause profuse or chronic diarrhea in humans and animals that may be life-threatening in immunocompromised people. Both pathogens are transmitted by the fecal-oral route to a broad range of hosts by direct contact or ingestion of contaminated food or water (Xiao, 2004). To date, thirty-nine *Cryptosporidium* species have been identified, and about 60 genotypes have been described, in various hosts and environmental samples (Feng et al., 2018; Holubová et al., 2019). More than 20 *Cryptosporidium* species or genotypes have been reported in human infections, of which about two-thirds originated from wild, free-range animals (Ortega, 2013). *Enterocytozoon bieneusi* is one of the 17 microsporidia reported to cause infections in humans and is the most frequently identified species in humans (Matos et al., 2012; Weiss and Becnel, 2015). The over 380 genotypes that have been identified are classified into eleven genetic groups (Li et al., 2019).

The Père David's deer (*Elaphurus davidianus*) is native to China and was once widely distributed in East Asia, but is now extinct in the wild because of hunting and reclamaton of swamp land in the late 19th century (Cao, 1985; Ohtaishi and Gao, 2010). About 3,000 Père David's deer live in captivity in China (Zhang et al., 2015). *Cryptosporidium ubiquitum*, *Cryptosporidium* deer genotype, and *E. bieneusi* genotypes IV, EbpC, EbpA, BEB6, COS–I, and COS-II have been identified in Père David's deer in Jilin and Henan, China (Zhang et al., 2015; Huang et al., 2018). The National Nature Reserve of Père David's deer in Shishou, Hubei Province is the major wildlife habitat of Père David's deer in China. About 1000 individuals live in the Nature Reserve. However, no report is available regarding the infection status of these pathogens in this deer populations. The aim of this study was to estimate the prevalence and molecular characterization of *Cryptosporidium* and *E. bieneusi* from Père David's deer in the National Nature Reserve of Shishou, Hubei Province, China.

## 2 Material and methods

### 2.1 Collection of fecal samples

The National Nature Reserve of Shishou, Hubei Province, China is located at the angle between the Changjiang River and the Swan Oxbow of the Changjiang River, with the geographic center coordinate being east longitude 112°23′ and north latitude 29°49’. The reserve covers an area of 1,567 ha and Père David's deer is the main protected object, with birds being the most abundant vertebrates. A total of 128 stool samples were obtained from Père David's deer within one week in July 2018 in the reserve. The formed stool samples were collected immediately after defecation using sterile disposable latex gloves and were transferred to individual plastic bags. All stool samples were collected with the assistance of experienced staff of the Nature Reserve.

### 2.2 Polymerase chain reaction (PCR) amplification

Genomic DNA was extracted from fecal samples using E.Z.N.A. Stool DNA kits (Omega Biotek Inc., USA). *Cryptosporidium* spp. were genotyped by nested PCR amplification and sequencing of the small subunit (SSU) rRNA gene (the primary primers SSU-F2 [5′-TTC TAG AGC TAA TAC ATG CG-3’] and SSU-R2 [5′-CCC ATT TCC TTC GAA ACA GGA-3’] and the secondary primers SSU-F3 [5′-GGA AGG GTT GTA TTT ATT AGA TAA AG-3’] and SSU-R4 [5′-CTC ATA AGG TGC TGA AGG AGT A-3’]) (Xiao et al., 2001). *C. parvum* subtyping was performed using the 60-kDa glycoprotein (*gp60*) gene (the primary primers AL3531 [5′- ATA GTC TCC GCT GTA TTC-3’] and AL3535 [5′-GGA AGG AAC GAT GTA TCT-3’] and the secondary primers AL3532 [5′-TCC GCT GTA TTC TCA GCC-3’] and AL3534 [5′-GCA GAG GAA CCA GCA TC-3’]) (Alves et al., 2003). *Enterocytozoon bieneusi* was identified by the presence of the internal transcribed spacer (ITS) gene (the primary primers AL4037 [5′-GAT GGT CAT AGG GAT GAA GA GCTT-3'] and AL4039 [5′-AAT ACA GGA TCA CTT GGA TCC GT-3'] and the secondary primers AL4038 [5′-AGG GAT GAA GAG CTT CGG CTC TG-3'] and AL4040 [5′-AAT ATC CCT AAT ACA GGA TCA CT-3']) (Sulaiman et al., 2003). Each 50 μL PCR mixture contained 1 × PCR buffer, 1.5 mM MgSO_4_, 0.2 mM dNTPs, 1 U KOD Plus (Toyobo Co. Ltd, Osaka, Japan), 1 μM of each primer. Positive (previously confirmed DNA sample) and negative controls (distilled water) were included in each PCR assay. PCR products were visualized by electrophoresis on 1% agarose gels (w/v) by GelRed™ (Biotium Inc., Hayward, CA, USA) staining.

### 2.3 Sequencing and phylogenetic analysis

PCR products were sequenced by GENEWIN (Suzhou, China); accuracy was confirmed by two-directional sequencing. The nucleotide sequences of each gene were aligned with GenBank reference sequences using ClustalX 2.1 (http://www.clustal.org/clustal2/) and manual adjustment. Phylogenetic analysis was performed by MEGA version 10 (https://www.megasoftware.net), with neighbor-joining trees constructed using the Kimura-2-parameter model to derive evolutionary distances between sequences. A total of 46 *E. bieneusi* genotypes with the nucleotide length of 300 bp to 450 bp were selected and used in the phylogenetic analysis. The *Cryptosporidium* SSU rRNA and *gp60*, and *E. bieneusi* ITS sequences have been deposited in the GenBank database under the accession numbers MK121773 to MK121777.

## 3 Results and discussion

PCR confirmed *Cryptosporidium* spp. infection in two of the 128 fecal samples (1.6%), lower than 6.4% (3/47) (χ^2^ = 2.88; *P* > 0.05) of prevalence reported in Père David's deer in Yuanyang County Forest Farm, Henan Province, China (it is worth mentioning that no *Cryptosporidium*-positive sample was detected in the same deer populations in 2008) (Wang et al., 2008; Huang et al., 2018). *Enterocytozoon bieneusi* was identified in 35.2% (45/128) the samples, which is similar to the 34.0% (16/47) prevalence reported in a previous study (Zhang et al., 2015).

*Cryptosporidium parvum* (n = 1) and *Cryptosporidium* deer genotype (n = 1) were identified by sequencing of the SSU rRNA gene. Previously, *C. ubiquitum* and *Cryptosporidium* deer genotype have been detected in Père David's deer in Henan, China (Huang et al., 2018). The *Cryptosporidium* deer genotype is host-specific but its prevalence within the deer population is reported to be low (Robinson et al., 2011; Wells et al., 2015; Kotkova et al., 2016). Nevertheless, the *Cryptosporidium* deer genotype has been found in white-tailed deer in the USA, and in the Czech Republic (Xiao et al., 2002; Santin and Fayer, 2015), roe deer in the UK (Robinson et al., 2011), red deer in China, and the UK (Wells et al., 2015; Huang et al., 2018), sika deer in Japan, and China (Kato et al., 2016; Huang et al., 2018), and Pere David's deer in China (Huang et al., 2018). *C. parvum* has not previously been reported in Père David's deer, although it has been found in red deer and roe deer in the UK, red deer in the Czech Republic, and white-tailed and black-tailed deer in the USA (Deng and Cliver, 1999; Perz et al., 2001; Hajdušek et al., 2004; Wells et al., 2015). Sequence analysis of gp60 gene found that the *C. parvum* isolate was the IIdA20G1 subtype, which has previously been found in human infections in Egypt, Iran, Kuwait, and Sweden (Gherasim et al., 2012; Helmy et al., 2013; Sulaiman et al., 2005; Taghipour et al., 2011), and buffalos and dairy cattle in Egypt, Sweden, and China ( Amer et al., 2013; 2010; Helmy et al., 2013; Mahfouz et al., 2014; Tao et al., 2001), and lambs in Romania (Imre et al., 2013). Père David's deer may thus be a source for *Cryptosporidium* infection in humans and other animals. In general, *C. parvum* IId is the most common subtype identified in China, including IIdA15G1 in rodents, cattle and yaks (Cui et al., 2014; Huang et al., 2014; Qi et al., 2015), IIdA18G1 in yaks (Qi et al., 2015), and IIdA19G1 in dairy cattle, humans, goats, yaks, and urban wastewater (Li et al., 2012; Mi et al., 2014; Qi et al., 2015; Wang et al., 2011, 2013), and IIdA20G1 in dairy cattle ( Tao et al., 2001).

**Fig. 1:**
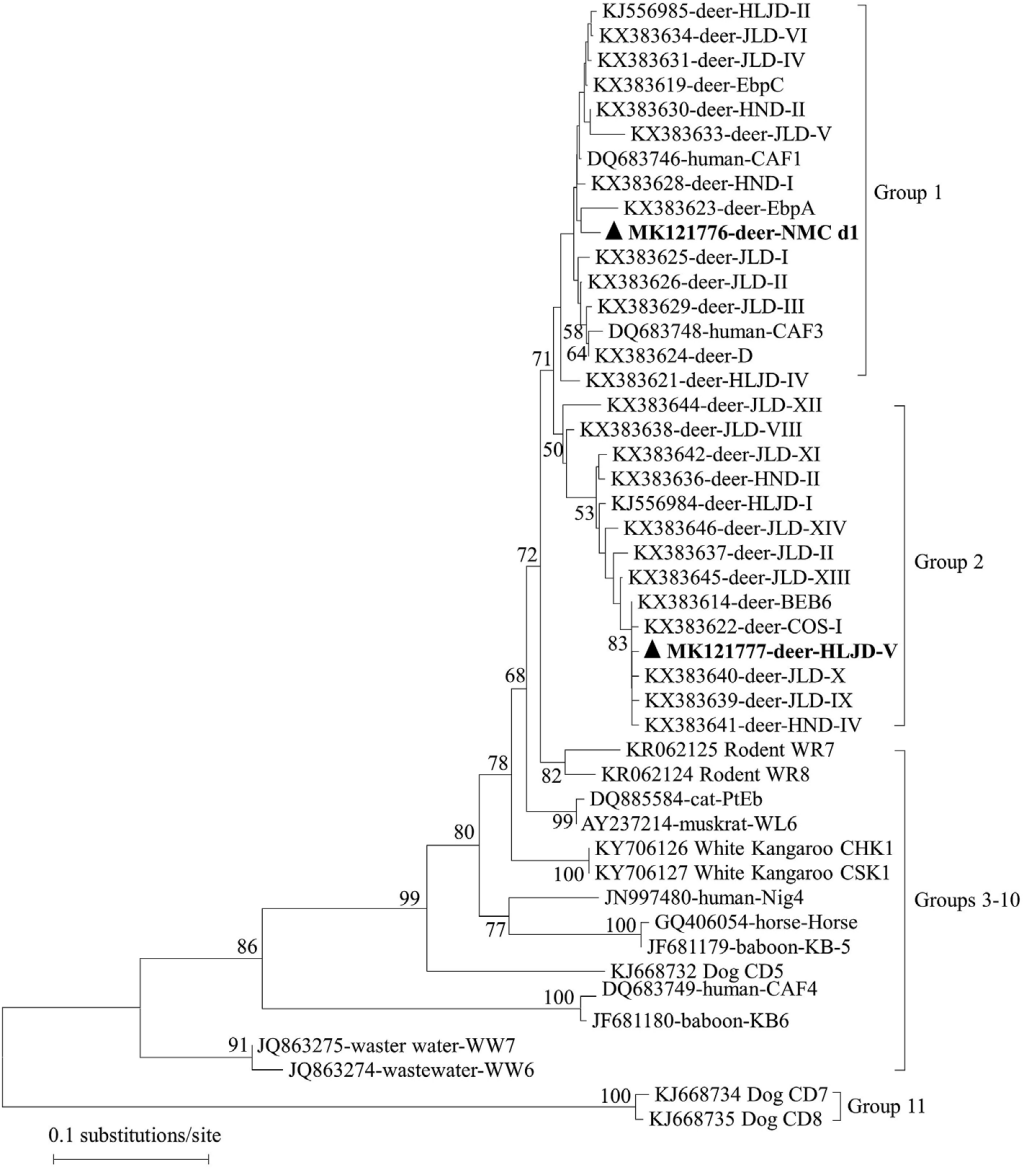
A phylogeny tree of the ITS sequences with distances calculated by neighbor-joining analysis using the Kimura two-parameter model. Bootstrap values > 50% from 1,000 replicates are shown on the nodes. The genotypes identified in this study are shown as triangles.

Sequence analysis of the PCR amplicons revealed the presence of two known *E. bieneusi* ITS genotypes, HLJD-V (n = 43), and MWC_d1 (n = 2) for the first time in Père David's deer. The HLJD-V genotype has been found in Sika and Red deer (Zhao et al., 2014), and MWC_d1 has been reported in Samber deer (Zhang et al., 2018). PCR assay of the ITS gene previously identified *E. bieneusi* Type IV, EbpC, EbpA, BEB6, COS–I, and COS-II in Père David's deer in Henan Province, China (Zhang et al., 2015). As shown in the Fig. 1, phylogenetic analysis found that genotypes MWC_d1 and HLJD-V were clustered with groups 1, and 2, respectively. The zoonotic potential of genotypes MWC_d1 and HLJD-V cannot be ignored. Genotypes in group 2 previously considered ruminant-adapted, however genotypes BEB4, BEB6, I and J, have been found in humans in the Czech Republic and China, indicating a possible risk of zoonotic infection (Sak et al., 2011; Wang et al., 2013). Nevertheless, multilocus sequence typing (MLST) will be better to estimate the transmission of *Enterocytozoon* genotypes identified in this study. Indeed, although molecular tools have indicated the potential for *Enterocytozoon* spp. from cervids to pose a threat to public health, as tools become more discriminatory, our current understanding may require revision (Robertson et al., 2019).

In conclusion, *E. bieneusi* infection was common in Père David's deer in China. Genotype HLJD-V was predominant in the study area. This is the first demonstration of *C. parvum* IIdA20G1 subtype in Père David's deer. These data suggest that the Père David's deer were infected with host-specific and/or zoonotic genotypes of these pathogens, implicating Père David's deer could be a potential source of human *Cryptosporidium* infection.

## Conflicts of interest

All authors declare no conflicts of interest.
